# Cheminformatic models based on machine learning for pyruvate kinase inhibitors of *Leishmania mexicana*

**DOI:** 10.1186/1471-2105-14-329

**Published:** 2013-11-19

**Authors:** Salma Jamal, Vinod Scaria

**Affiliations:** 1 CSIR Open Source Drug Discovery Unit, Anusandhan Bhavan, New Delhi, 110001, India; 2 GN Ramachandran Knowledge Center for Genome Informatics, CSIR Institute of Genomics and Integrative Biology, Mall Road, Delhi, 110007, India

## Abstract

**Background:**

Leishmaniasis is a neglected tropical disease which affects approx. 12 million individuals worldwide and caused by parasite *Leishmania.* The current drugs used in the treatment of Leishmaniasis are highly toxic and has seen widespread emergence of drug resistant strains which necessitates the need for the development of new therapeutic options. The high throughput screen data available has made it possible to generate computational predictive models which have the ability to assess the active scaffolds in a chemical library followed by its ADME/toxicity properties in the biological trials.

**Results:**

In the present study, we have used publicly available, high-throughput screen datasets of chemical moieties which have been adjudged to target the pyruvate kinase enzyme of *L. mexicana* (LmPK). The machine learning approach was used to create computational models capable of predicting the biological activity of novel antileishmanial compounds. Further, we evaluated the molecules using the substructure based approach to identify the common substructures contributing to their activity.

**Conclusion:**

We generated computational models based on machine learning methods and evaluated the performance of these models based on various statistical figures of merit. Random forest based approach was determined to be the most sensitive, better accuracy as well as ROC. We further added a substructure based approach to analyze the molecules to identify potentially enriched substructures in the active dataset. We believe that the models developed in the present study would lead to reduction in cost and length of clinical studies and hence newer drugs would appear faster in the market providing better healthcare options to the patients.

## Background

Leishmaniasis is a tropical disease affecting 12 million people worldwide, with approximately ~2 million (1.5 million incidences of cutaneous leishmaniasis and 500,000 visceral leishmaniasis) new people getting infected each year [1]. It is considered as one of the world’s most neglected disease given its strong association with poverty and limited resources invested in new tools for diagnosis, treatment, and control [2]. Among tropical diseases, leishmaniasis ranks second as a causative factor in mortality and fourth in morbidity and has been reported to occur in as much as 88 countries. It affects massive populations in most tropical and subtropical regions resulting in a huge number of deaths. The disease has become a major threat to one-third of the world population with more than 90% of the cases arising out of India, Bangladesh, Sudan, South Sudan, Brazil and Ethiopia [3-5]. Leishmaniasis is caused by a Trypanosomatid protozoan parasite belonging to the genus *Leishmania,* which infect both human and domestic animals, resulting in significant social and economic losses, especially in developing nations [6]. The infection spreads through the bite of the phlebotomine sandflies which injects the promastigotes into the host [7]. Approximately 21 of 30 species cause infections in humans and include *L. donovani* complex with three species (*L. donovani, L. infantum*, and *L. chagasi*); the *L. mexicana* complex with four main species (*L. mexicana, L. amazonensis*, and *L. venezuelensis*); *L. tropica; L. major; L. aethiopica*; and the subgenus *Viannia* with four main species (*L. (V.) braziliensis, L. (V.) guyanensis, L. (V.) panamensis*, and *L. (V.) peruviana*) [8]. *L. Mexicana* causes both cutaneous and diffused cutaneous types of infection [9]. The disease is considered as a major constraint to economic development with symptoms ranging from self-healing ulcers to highly disfiguring lesions and serious, often lethal visceral diseases which affect the haemopoetic organs [10].

The therapy of Leishmaniasis has been quite a challenge given the fact that the commonly used drugs available for treatment are characterized by high toxicity, high costs, limited activity and considerable possibility of drug resistance [11,12]. The first line drugs used in the therapy are antimonial compounds such as sodium stibogluconate and meglumine antimoniate which form the traditional therapy for leishmaniasis. They are administered through the parenteral route and have severe side effects. In case of failure of the first line drugs, second line drugs are used, which include pentamidine (Lomidine) and amphotericin B (Fungizone). However, both these drugs are also associated with high levels of toxicity and side effects. Similarly, the new drug, Miltefosine (Impavido) prescribed for visceral and cutaneous leishmaniasis has also been identified to cause adverse effects [13,14]. It has a long residence time which may contribute to the selection of resistant parasites, limiting its applicability. Miltefosine stays for a longer duration time in circulation approximately 150 hours which may lead to the development of resistance owing to which the parasite spreads rapidly [15]. The current approach based on chemotherapy relies on a handful of drugs which are limited by factors such as high costs, toxicity, difficult routes of administration, and less efficacy [16]. Keeping in mind all these factors, it is necessary to develop reasonably priced, secure, and effective antileishmanial vaccines for the acceptable therapy of leishmaniasis.

In *Leishmania sp.*, sugar uptake and gluconeogenesis are essential to synthesize hexose-phosphates necessary for the production of glycoconjugates and the intracellular polysaccharide mannan, which form essential components for both replication and virulence of the parasite [17]. Therefore, glycolytic enzymes are extremely pertinent for the growth and infectivity of the parasite.

Glycolysis pathway and enzymes in the pathway has been extensively reviewed as a potential drug target candidates [18]. One of the well characterized enzymes in the pathway is Pyruvate kinase, which also has been extensively studied as a candidate drug target. Trypanosomatids entirely depend on the carbon sources available inside the host to meet their energy requirements and the only source of ATP generation is glycolysis as they lack Kreb’s cycle. Pyruvate kinase plays an important role in carbohydrate and amino acid metabolism and catalyses the last step in glycolysis to produce ATP and pyruvate kinase. Several reports have exploited the features of glycolytic enzymes on the basis of the enzyme’s structure further leading to the utilization of these features for the design of specific inhibitors [19-21]. Leishmania mexicana is known to encode for two copies of the enzyme, organized tandem to one another [19]. The crystal structure of the enzyme has also been elucidated recently [22]. The recent availability of high throughput screens for drug discovery of neglected diseases has motivated us to create predictive models based on molecular properties and machine learning approaches [23-26]. Recently a large dataset of high-throughput screens have been made available in public domain for Leishmania Mexicana Pyruvate Kinase and forms the baseline for the present study.

In the present study, we have used a computational strategy to create predictive classification models from the high-throughput assay which target pyruvate kinase enzyme from *L. mexicana* (LmPK). We have further analyzed chemical substructures to find enriched bioactive molecules using Maximum Common Substructure (MCS) approach and we also show that machine learning based cheminformatic modeling can create predictive models with high accuracy which can be effectively used to screen large molecular databases in silico, thus drastically reducing the cost of finding hits for drug discovery.

## Methods

### Bioassay and data sources

The assay used in the current study targets pyruvate kinase from *Leishmania mexicana* (LmPK). The datasets for the assay have been deposited at PubChem, a database collecting information on small molecules and datasets on high throughput biological assays and maintained by the National Centre for Biotechnology Information (NCBI) [27]. The assay [AID: 1721] consisted of a total of 292,740 compounds capable of inhibiting the enzyme, pyruvate kinase, derived from *Leishmania mexicana.* Compounds were characterized based on a PubChem activity score. The compounds that had an activity score between 40 and 100 were defined as active (N = 1,087) and the compounds having an activity score of 0 were defined as inactive (N = 289,657). All the compounds having activity score between 1 and 39 were considered to have inconclusive activity and were not included in our analysis in order to avoid uncertainty in the predictive ability of the models.

### Dataset pre-processing and calculation of molecular descriptors

The chemical structure of each of the molecules was downloaded in the Structural Data Format (SDF) from PubChem. These structures were imported into the molecular descriptor generator and visualization software PowerMV [28]. PowerMV generates 2D molecular descriptors and is freely available. The dataset was split into smaller SDF files using SplitSDFiles Perl script available from Mayachem tools [29]. A total of 179 descriptors were computed for the molecules. These descriptors encompassed different categories and included 147 descriptors which were pharmacophore fingerprints, 24 descriptors which were weighted burden numbers and 8 which belonged to property descriptors. The bit-string fingerprint attributes of only one value (all 0’s or all 1’s) all across the molecules were removed to reduce the dimensionality of the dataset. The full set of compounds were randomly divided into 20% independent test set and 80% training cum validation set using a bespoke Perl script. We used 5-fold cross validation in our study.

### Machine Learning methods and implementation

Machine learning is a scientific discipline that broadly refers to a collection of algorithms and computational methods for predictive learning from tagged datasets [30]. In cheminformatics, such methods have been extensively used to predict molecular properties, or biological activities. Generally, molecular datasets are tagged on the basis of their activity; say active/inactive and binary classification based on a set of molecular descriptions could be attempted. We have earlier shown that such an approach could accurately predict the activities in diverse sets of datasets with activities as diverse as anti-tubercular [23,24] molecules, anti-malarial molecules [25] and RNA-binders [26]. Similar tagging and learning could be attempted for multiple classes, rather than binary sets and have been extensively reviewed in [31]. Multiple algorithms and implementations have been used in the area previously; nevertheless we attempted four popular classifier algorithms, that is, Naïve Bayes, Random Forest, J48 and SMO. All four methods have been previously determined to be quite efficient in terms of both computation time and classification accuracies. The Naive Bayes classifier is based on the Bayesian theorem, which assumes that for a given target value, the description of each predictor is independent of the other predictors. The final prediction is obtained by considering all descriptor-based properties [32]. Random Forest algorithm is based on decision trees, where each tree is independently constructed and each node is split using the best among a subset of predictors randomly chosen at the node. It is the most accurate classifier and produces most precise results for all the datasets [33]. J48 is a version of an earlier algorithm, the very popular C4.5, developed by J. Ross Quinlan and employs a tree pruning approach which produces fewer but more easily interpreted results. The J48 algorithm chooses one attribute of the data and splits the set of samples into subsets, one for every value of the attribute. The attribute having the maximum information gain is chosen to make the decision [34]. Sequential Minimization Optimization (SMO) algorithm developed by John Platt in 1998 is widely used for training support vector machines. SMO, an iterative algorithm, breaks up the quadratic programming (QP) optimization problem into smaller problems which are then solved analytically. The SMO algorithm is simple, easy to use and faster in comparison to the standard SVM training algorithm [35].

### Cost sensitive classification

One of the key issues that needs to be taken into consideration while using machine learning technique on a highly imbalanced dataset is the cost of misclassification. This is an important issue because standard classifiers presume equal weighing for all the classes and thus are unable to handle imbalanced data [36]. The use of cost-sensitive classifiers can abrogate this issue and minimize misclassification errors. In cost sensitive learning, misclassification costs are used in which molecules are predicted to have the class with lower expected cost [37].

In the present study, we have used Weka (Waikato Environment for Knowledge Analysis), a collection of machine learning algorithms, for data mining tasks [38]. Weka uses a confusion matrix consisting of four sections: True Positives (TP) for correctly classified actives; False Positives (FP) for inactive classified as actives; True Negatives (TN) inactive classified as inactive and false negatives (FN) for active compounds incorrectly classified as inactive. One of the most important points to be well thought-out during the development of classifiers is the fact that the false negatives are considered to be more important than the false positives. Consequently, we can minimize the % of false negatives at the expense of increasing false positives. To keep a check on the rate of false positives, an upper limit of 20% is set on the false positives. In Weka, there are no rules to set misclassification cost. It exclusively depends on the base classifier used [39].

### Statistical measures for evaluation of cheminformatics models

A variety of measures were used to evaluate the performance of models such as sensitivity, specificity, accuracy, and BCR. Sensitivity (TP/(TP + FN)) is the proportion of positively labeled molecules predicted correctly. Specificity (TN/(TN + FP)) is the percentage of negatively labeled instances predicted as negative. Accuracy ((TP + TN)/(TP + TN + FP + FN) * 100) is the percentage coverage of correct predictions. Balanced Classification Rate (BCR) (½. (Sensitivity + Specificity)) is the mean of sensitivity and specificity which introduces a balance amid the classification rate of the two classes. Matthews Correlation Coefficient (MCC) is regarded as the balanced measure that measures the quality of a binary classification. We also evaluated the models based on the Receiver Operating Characteristic (ROC) curve which is the plot between the true positive rate and false positive rate.

### Evaluation of enriched substructures

We used a hierarchical clustering algorithm ‘LibMCS’ , available from Chemaxon to find out potentially enriched molecular substructures in bioactive molecules [40]. The maximum common substructure (MCS) based approach retrieves and compares the substructure common to a group of molecules. The MCS size which corresponds to the number of constituent atoms was set to an empirical threshold of 14 atoms in this study. The scaffolds obtained were then used to search for similar molecules in active and inactive datasets using the ‘jcsearch’ algorithm available from Chemaxon [41]. The Chi-square test and the associated p-value were used for the further evaluation of substructures and to test the significance of enrichment, respectively. We calculated the enrichment factor and used a threshold of 2 to prioritize the molecules for further analysis. Using vROCS (release 3.1.2) [42], we further performed a molecular alignment of the selected scaffolds with the molecules in the active dataset and visualized the alignment in VIDA software [43] available from Open Eye Scientific Software [44].

## Results and discussion

The dataset of active (1,087) and inactive (289,657) molecules was downloaded from PubChem. A total of 179 2D molecular descriptors were generated using PowerMV for the entire set of molecules. After the pre-processing of data (as described in Methods), the number of molecular descriptors was further pruned to 154, which accounted for an approximate 15% reduction in the number of descriptors. To begin with, the standard classifiers were used to generate the models, however, cost sensitive classification was used in case of models having low FP rate and the cost was increased for FP up to 20%. The final mis-classification cost used for each classifier is presented in Table 1. The Naive Bayes classifier required lowest misclassification cost and was quite fast in terms of compute time.

**Table 1:** Classification results

**Classifier**	**TP rate**	**FP rate**	**ROC**	**Accuracy**	**BCR**	**MCC**	**Cost**
NB	72.4	19.7	84	80.26	76	0.08	60
RF	87.1	20	91.3	80.01	83	0.10	80000
J48	82	20.2	80.5	79.76	80	0.09	1200
SMO	85.7	19.2	89.8	80.79	82	0.10	250

**Figure 1:**
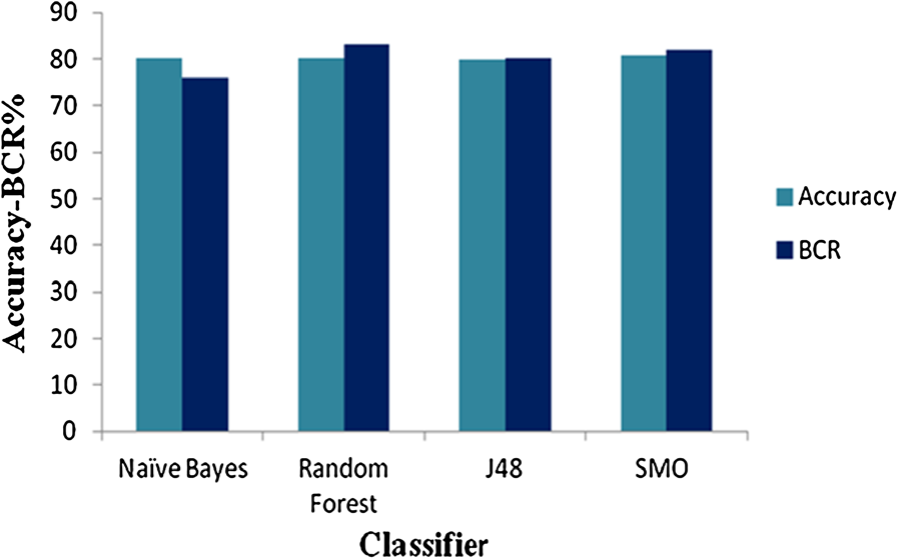
Comparison of Accuracy and Balanced Classification Rate of the models generated in the present study.

**Figure 2:**
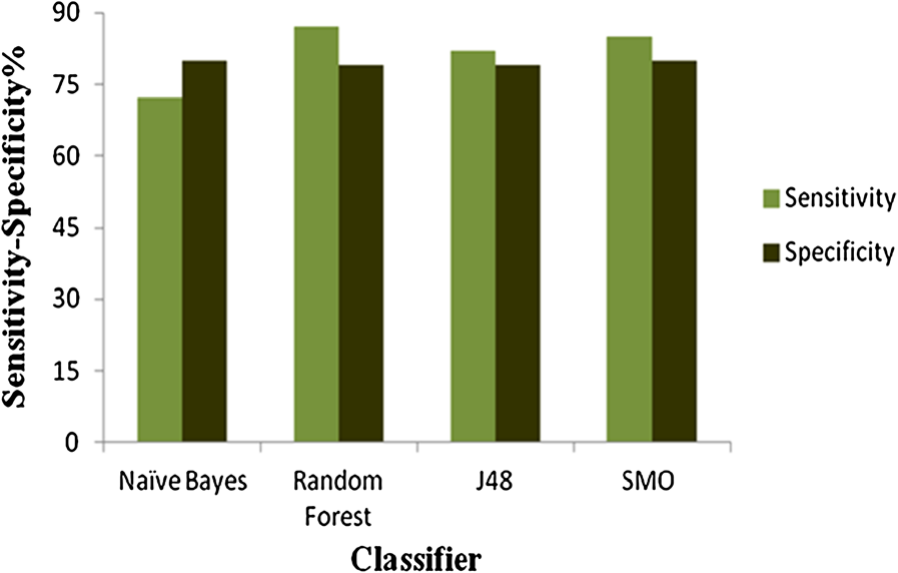
Plot of Sensitivity and Specificity of models generated based on molecular descriptors.

**Figure 3:**
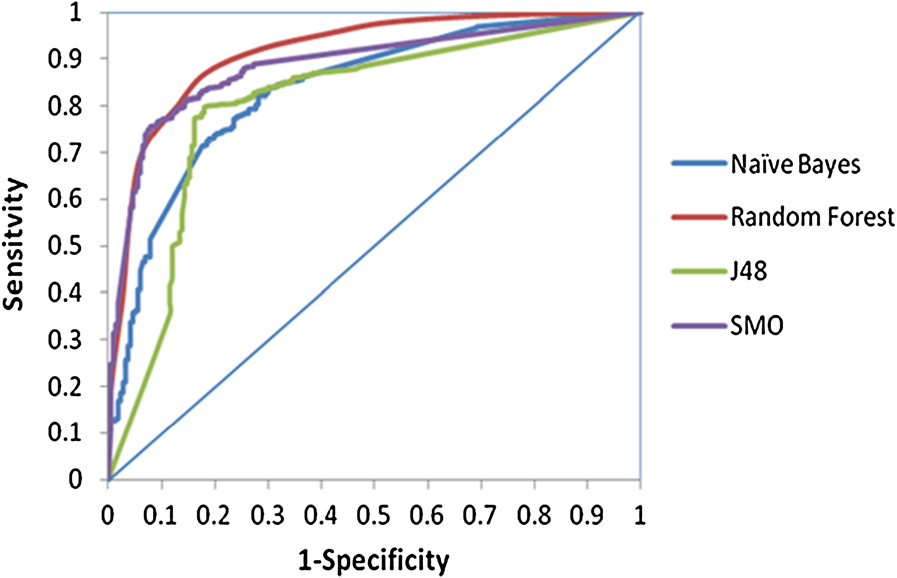
ROC plot showing significant AUC values for Random Forest, Naive Bayes, J48 and SMO classifiers.

A number of models were generated using different classifiers described in the materials and methods section. The best models for each classifier were selected on the basis of accuracy of the models generated. In the present study, all the models generated had around 80% accuracy (Figure 1). Various other statistical figures such as sensitivity, specificity and BCR were also used to check the robustness of the models. Since accuracy alone cannot be used to assess the performance of the models owing to the high imbalance in the data, we have used Balanced Classification Rate (BCR) which introduces a correct balance in the sensitivity and specificity and gives a more accurate measure of the performance of the models. All the models had around 80% sensitivity and specificity with the RF model being the most sensitive and NB the least (Figure 2). Also the RF model turned out to be the most accurate classifier having BCR, the average of sensitivity and specificity, value as 83%. We also performed an analysis of the Receiver Operator Characteristics (ROC) which was further used to compare and evaluate the performance of each of the models for their efficiency and robustness. All the models had a significant Area under Curve (AUC) on the ROC plot, which can be seen in Figure 3. It can be easily interpreted from the results that among all the classifiers, i.e. NB, RF, SMO and J48, Random Forest performed better than the rest and was established as the best classifier providing an overall good classification.

**Figure 4:**
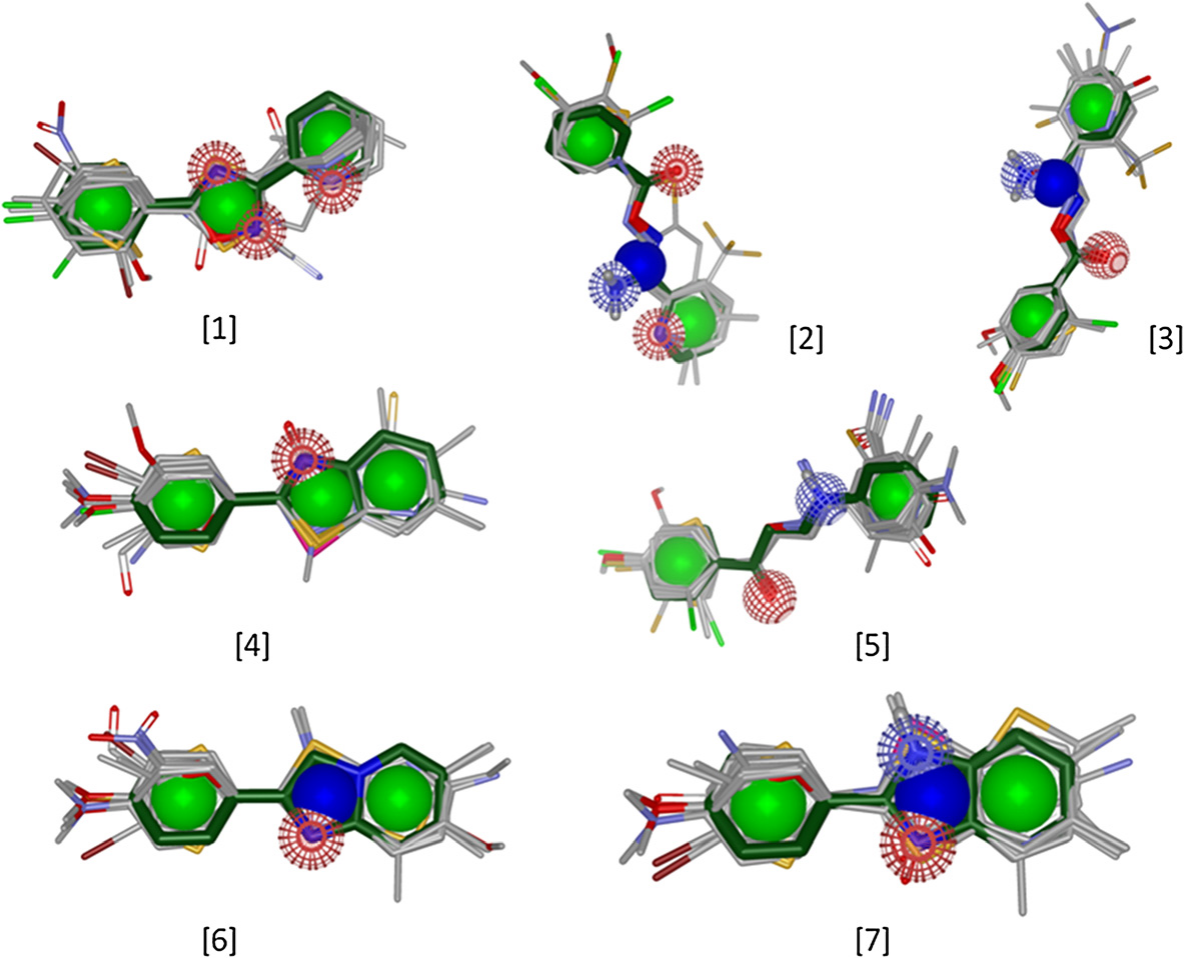
**Molecular alignment of the 7 [**[1]**-**[7]**] enriched substructures (dark green) over the top 20 molecules of the active (1087) dataset obtained from PubChem (AID 1721).**

We further evaluated whether we could understand the common or frequent molecular substructures which were associated with the molecular activity. To this end, all the active dataset compounds were clustered using LibMCS algorithm. We obtained a total of 3,418 substructures clustered up to 6 levels. A total of 501 clusters at level 6 were selected, from which 331 singletons were separated. We calculated the Chi-square and p-value for the remaining 170 substructures which correspond to the clusters were analyzed for enrichment and its significance in the active and inactive datasets (Table 2). The substructures with a frequency of >1% in the active dataset were taken that accounted for a total of 10 substructure. Stringent filtering retrieved a total of 7 substructures which had p-values less than 0.01 and enrichment factor >2. We did the molecular alignment of the selected 7 enriched substructures with the active molecules (Figure 4) and inactive molecules to calculate the enrichment of the scaffolds between the active and inactive datasets.

**Table 2:** Significantly enriched substructures in the active dataset

**Serial no.**	**Scaffold**	**Actives**	**Inactives**	**Chi-square**	**p-value**	**Enrichment factor**
Scaffold 1		12	3	2553.48	0.00E + 00	1065.89
Scaffold 2		13	29	1054.51	0.00E + 0	119.45
Scaffold 3		11	82	327.68	3.08E-73	35.74
Scaffold 4		12	143	226.03	4.36E-51	22.36
Scaffold 5		19	234	346.19	2.86E-77	21.36
Scaffold 6		18	223	325.98	7.22E-73	21.50
Scaffold 7		12	271	113.69	1.52E-26	11.79

The recent years have seen a wealth of information being available in public domain on molecular structures and biological assays of very small subsets of known small molecule repertoire using high-throughput screening platforms. The major challenge within the field pertains to the assigning potential biological activities to molecules so that they could be studied in detail. On an average less than a percent of the molecule library screened show some biological activity. Given the large costs associated with setting up screens for biological activities, it would be economically not plausible to exhaustively screen large parts of the known small molecule repertoire known to mankind. This problem becomes acute in cases of neglected tropical diseases. The challenge therefore would be to effectively mine large libraries using computational tools so they could be effectively prioritized for experimental screening for their biological activities. This necessitates the creation of highly accurate computational methods capable of predicting biological activities in silico. In the present study, we use machine learning as an approach to build highly accurate predictive models for bio-activity against pyruvate kinase on Leishmania species. We show how high-throughput experimental datasets on a diverse set of molecules could potentially be used to build highly accurate predictive models. These models could potentially be used to mine and annotate large molecular datasets and prioritize molecules for biological activity screening experiments and could contribute significantly to the ongoing efforts for drug discovery for neglected tropical diseases.

## Conclusion

Leishmaniasis is one of the major neglected tropical diseases in recent years, killing close to 100,000 individuals worldwide annually, mostly in the tropical and sub-tropical countries. The disease is majorly distributed in the tropical and sub-tropical regions. Though efficient treatment regimens are available for its therapy, the drugs used are largely toxic. In addition, wide-spread drug resistance has been reported in several regions, adding to the urgency for the discovery of novel, efficient and less-toxic molecules with anti-leishmanial activity. In this study, we have employed computational strategy via machine learning approach to create predictive models for classification of molecules to discover new therapeutic compounds for leishmaniasis. The approach will help in quick search of large libraries of chemical structures in order to pick potential hits which are most probable to bind to a drug target. Additionally, we have used a substructure based approach to explore potentially enriched substructures in the active dataset of molecules. We show that accurate models for mining large datasets could be built based on high throughput assays available in public domain using machine learning approaches. We have previously reported similar approaches for mining molecules with anti-tubercular activities, and suggest that Random Forest based learning can systematically learn from bioassay datasets with high accuracies. We argue that automated approaches based on Random Forest based algorithms could be implemented on a large-scale to learn from bioassay datasets and automatically annotate molecules from PubChem for biological activities. We hope that such approaches could accelerate the process and efficiency of screening for discovery of novel molecules with specific biological activities not just for tropical diseases but others as well.

### Model availability

The predictive model generated by Weka, a stepwise manual and the scripts to be used for preprocessing of the dataset are available online at http://vinodscaria.rnabiology.org/2C4C/models.

## Competing interests

The authors declare that they have no competing interests.

## Authors’ contributions

SJ under the supervision of VS carried out the analysis and reviewed the results. OSDDC supported the work through regular discussions and funding. Both authors wrote, reviewed and approved the final manuscript.
